# Supplementary material for: Light-induced giant enhancement of nonreciprocal transport at KTaO3-based interfaces
Source: Nat Commun. 2024 Apr 6;15:2992. doi: 10.1038/s41467-024-47231-6 (PMC10998845; doi:10.1038/s41467-024-47231-6)
Supplement: Supplementary file 1 — Supplementary Information [file 41467_2024_47231_MOESM1_ESM.pdf]

Supplementary Information

Light-induced giant enhancement of nonreciprocal transport at $KTaO_3$-based interfaces

Xu Zhang[1,8], Tongshuai Zhu[2,3,8], Shuai Zhang[2,8], Zhongqiang Chen[1], Anke Song[1], Chong Zhang[1], Rongzheng Gao[1], Wei Niu[1], Yequan Chen[1], Fucong Fei[2], Yilin Tai[4], Guoan Li[5], Binghui Ge[4], Wenkai Lou[6], Jie Shen[5], Haijun Zhang[2], Kai Chang[6], Fengqi Song[2,*], Rong Zhang[1,7,*] & Xuefeng Wang[1,*]

[1]Jiangsu Provincial Key Laboratory of Advanced Photonic and Electronic Materials, State Key Laboratory of Spintronics Devices and Technologies, School of Electronic Science and Engineering, Collaborative Innovation Center of Advanced Microstructures, Nanjing University, Nanjing 210093, China

[2]National Laboratory of Solid State Microstructures, School of Physics, Nanjing University, Nanjing 210093, China

[3]College of Science, China University of Petroleum (East China), Qingdao 266580, China

[4]Information Materials and Intelligent Sensing Laboratory of Anhui Province, Institutes of Physical Science and Information Technology, Anhui University, Hefei 230601, China

[5]Beijing National Laboratory for Condensed Matter Physics and Institute of Physics, Chinese Academy of Sciences, Beijing 100190, China

[6]State Key Laboratory for Superlattices and Microstructures, Institute of Semiconductors, Chinese Academy of Sciences, Beijing 100083, China

[7]Department of Physics, Xiamen University, Xiamen 361005, China

[8]These authors contributed equally: Xu Zhang, Tongshuai Zhu, Shuai Zhang

[*]**e-mail:** songfengqi@nju.edu.cn; rzhang@nju.edu.cn; xfwang@nju.edu.cn

Content

Supplementary Fig. 1. Basic structural characterization.
Supplementary Fig. 2. EDX elemental mapping and the corresponding spectrum.
Supplementary Fig. 3. Schematic illustration of the Hall-bar device fabrication process.
Supplementary Fig. 4. Homogeneity test and 2D conducting characteristic of the 2DEGs at the CZO/KTO interfaces.
Supplementary Fig. 5. Stability test of the 2DEGs at the CZO/KTO interfaces.
Supplementary Fig. 6. Current dependence of the nonreciprocal transport.
Supplementary Fig. 7. Temperature dependence of the nonreciprocal transport.
Supplementary Fig. 8. Second harmonic measurements for the nonreciprocal transport of KTO-based 2DEGs.
Supplementary Fig. 9. Hysteretic MR and R_{xy} measurements.
Supplementary Fig. 10. The magnetoconductance curves and the deduced other SOC-related parameters at various wavelengths at 5 K.
Supplementary Fig. 11. Hall curves perfectly fitted using the two-band model.
Supplementary Fig. 12. The physical mechanism of photocarrier excitation.
Supplementary Fig. 13. The band structures from first-principles calculations plus Hubbard *U*.

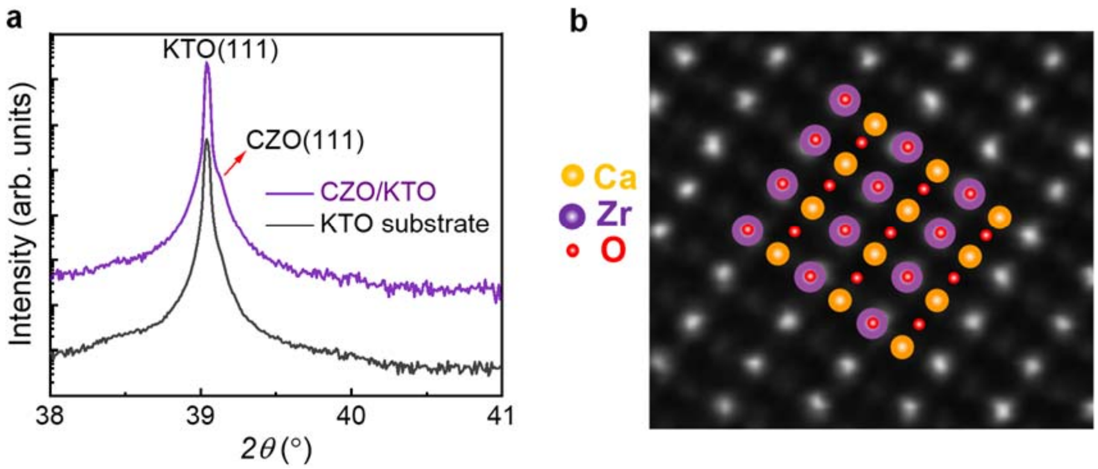

Supplementary Fig. 1. Basic structural characterization. a, θ-2θ x-ray diffraction (XRD) pattern for the CZO/KTO (111) sample. The diffraction peak of CZO can be traced from the KTO substrate, implying the excellent epitaxy. **b**, The enlarged atomic configuration overlapping on the STEM image, which is taken from **Fig. 1b**.

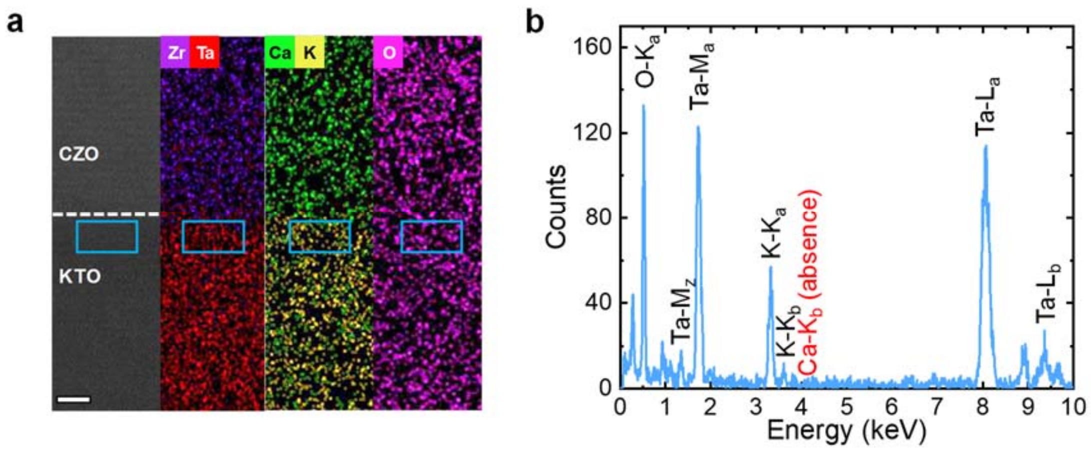

Supplementary Fig. 2. EDX elemental mapping and the corresponding spectrum. **a**, EDX elemental mapping at the interface. A quantitative spectroscopic analysis is conducted in the region within the blue boxed area. The white dashed line is a guide to indicate the interface. The scale bar is 5 nm. **b**, The corresponding EDX spectrum, where the characteristic peaks of K, Ta and O elements are clearly seen. However, no peaks of Ca element are detected, indicating the noticeable Ca element in the KTO substrate in **a** is actually an artifact due to the very close energy edges of Ca and K from EDX.

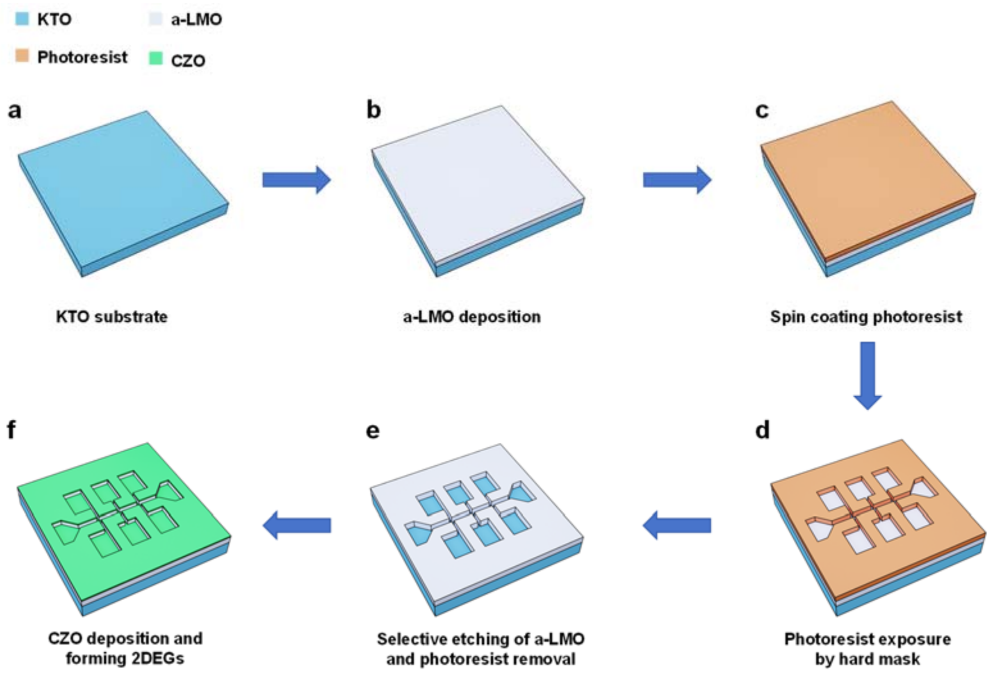

Supplementary Fig. 3. Schematic illustration of the Hall-bar device fabrication process. a-b, The amorphous $LaMnO_3$ (a-LMO) film is deposited by the PLD on the KTO substrate. **c**, The photoresist is spin-coated onto the a-LMO surface. **d**, Hall-bar pattern of photoresist is fabricated by the ultraviolet exposure. **e**, The selective etching by HCl/KI solution and the removal of the rest photoresist, forming the Hall-bar pattern of the a-LMO film. **f**, The CZO layer is deposited by PLD. The 2DEGs thus forms at the a-LMO-patterned CZO/KTO interfaces.

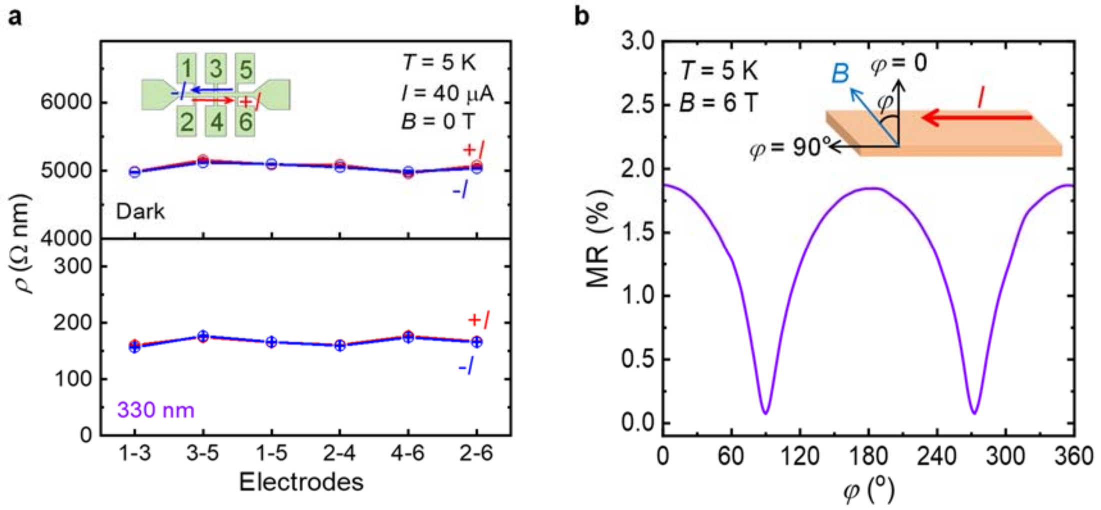

Supplementary Fig. 4. Homogeneity test and the 2D conducting characteristic of the 2DEGs at the CZO/KTO interfaces. a, The resistivity of the arbitrarily selected electrodes by applying both $I = \pm 40$ μA in the dark and under 330-nm illumination, respectively. The temperature is 5 K without the applied external field. The inset is the sketch of this Hall-bar electrodes for the homogeneity test. It can be seen that there is no significant difference of the resistivity between each electrode in both dark condition and under illumination, indicating the excellent homogeneity of the conducting channel of 2DEGs. Besides, no remarkable difference is observed when applying the opposite-direction current, further ruling out the inhomogeneity of the carrier distribution. The lines are drawn as guides for the eyes. **b**, The anisotropic MR as a function of the direction of the applied field at 5 K and 6 T for the CZO/KTO heterostructures. The inset shows the sketch of the anisotropic MR measurement. The sharp peaks at 90° and 270° when the field is applied parallel or antiparallel to the current indicate the 2D conduction characteristic and also the uniformity of the 2DEGs at the interface. The thickness of the conduction layer (t) can be estimated by adopting the formula of $t = h/(e^2 k_F R_s \sqrt{\alpha})$, where h is the Planck constant, e is the electron charge, k_F is the Fermi wave vector ($k_F = \sqrt{2\pi n_s}$), R_s is the sheet resistance, and α is defined by the ratio of the perpendicular MR to the parallel MR. The conduction layer thickness of KTO-based 2DEGs is thus estimated to be ~4.35 nm.

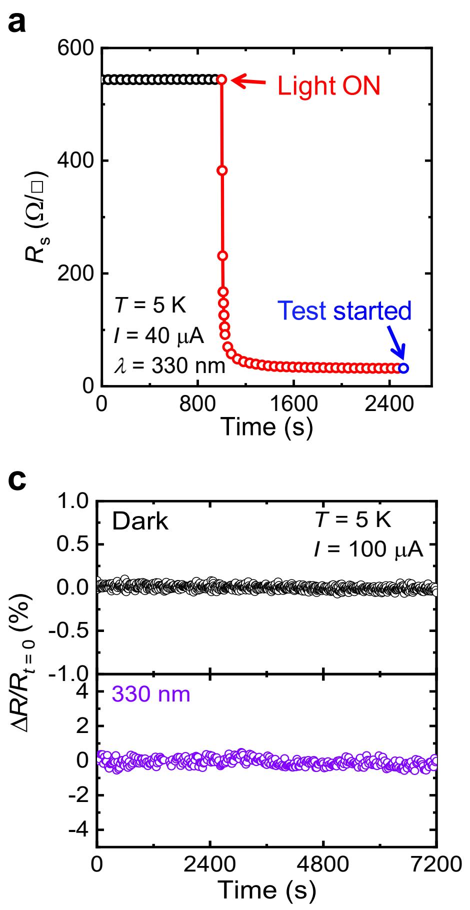

Supplementary Fig. 5. Stability test of the 2DEGs at the CZO/KTO interfaces. a, The time evolution of the sheet resistance under the in-situ light illumination at 330 nm at 5 K. The MR and Hall effect measurements were carried out when the sheet resistance is stabilized under the light illumination. **b**, The time evolution of the ratio of the resistance change ($\Delta R/R_{t=0}$) under 330-nm illumination at 5 K. $R_{t=0}$ represents the initial sheet resistance at t = 0. $\Delta R = R_t - R_{t=0}$ represents the resistance change. The applied current is 40 μA. A negligible resistance variation of less than 0.5% is observed with the time evolution for at least 2 h, excluding the light-induced heating effect. **c**, The time evolution of $\Delta R/R_{t=0}$ in the dark and under 330-nm illumination at 5 K, respectively. The applied current is as large as 100 μA. The resistance still maintains nearly unchanged (within 0.6%) even under 330-nm illumination at 5 K for at least 2 h, ruling out any light-induced/large-current-induced heating effect during the light-irradiation measurements. **d**, The time evolution of the resistance at the a-LMO/KTO interface under the light illumination at 330 nm at 5 K. It is seen that the a-LMO/KTO interface always maintains insulating (~10^6 Ω/□) during the illumination.

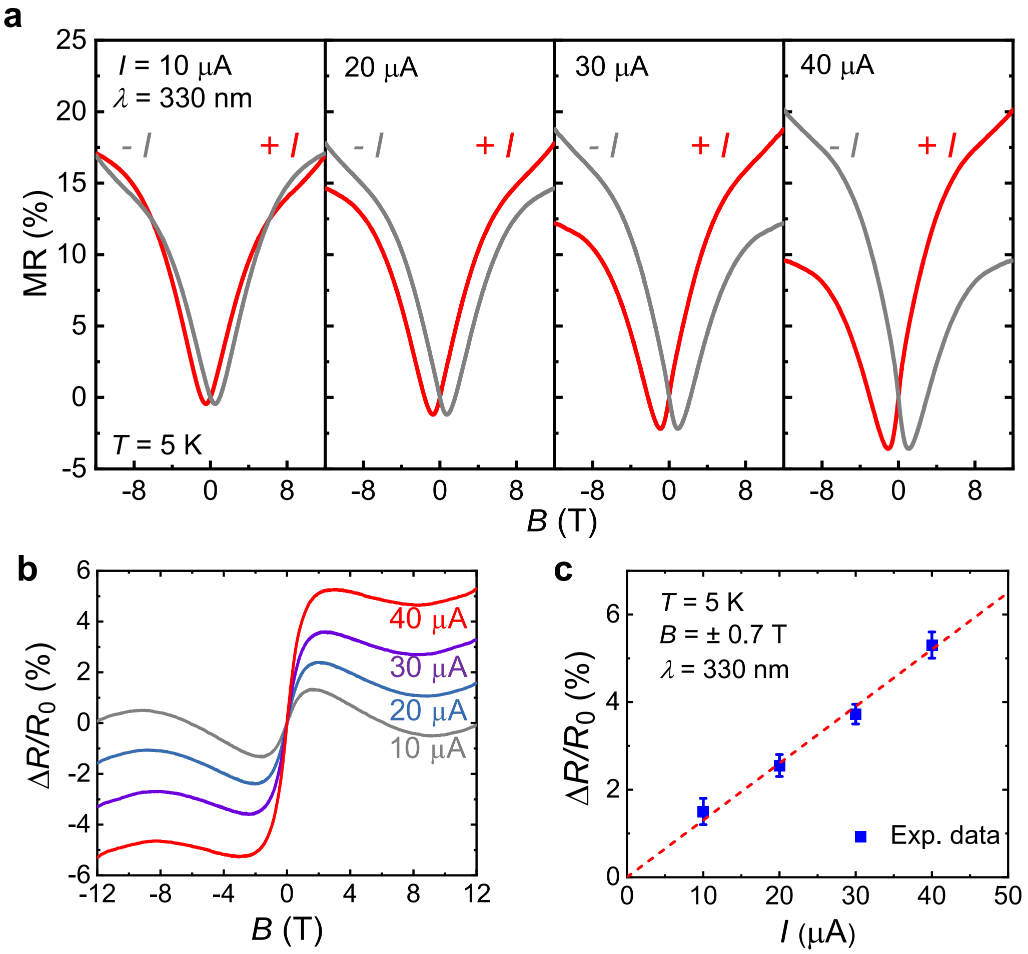

Supplementary Fig. 6. Current dependence of the nonreciprocal transport. a, In-plane MR curves measured at 330 nm with applied different currents at 5 K. **b**, The ratio of the resistance change ($\Delta R/R_0$ extracted from **a**) as a function of magnetic field at various currents. **c**, $\Delta R/R_0$ at ±0.7 T as a function of applied current. The red dashed line is a guide to the eye, indicating the linear behavior. The error bars indicate the uncertainty from the experimental results. The red dashed line is the linear fitting.

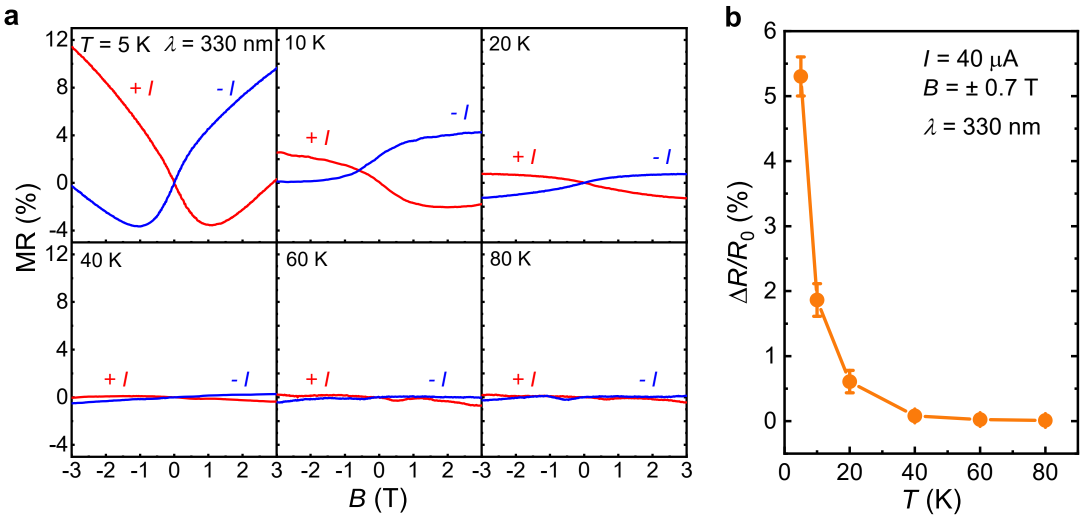

Supplementary Fig. 7. Temperature dependence of the nonreciprocal transport. **a**, In-plane MR curves measured at 330 nm under different temperatures. The applied current $I = \pm 40$ μA. **b**, The ratio of the resistance change ($\Delta R/R_0$ at ±0.7 T extracted from **a**) as a function of temperature. The nonreciprocal transport vanishes above 40 K, which is attributed to the disruption of spin-momentum locking by quantum fluctuations at relatively high temperatures. The error bars indicate the uncertainty from the experimental results. The line connecting the data points is drawn as a guide for the eyes.

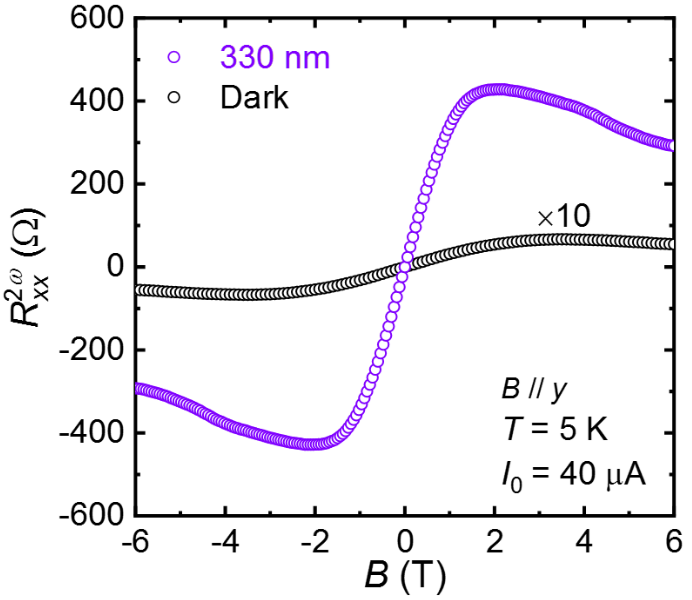

Supplementary Fig. 8. Second harmonic measurements for the nonreciprocal transport of KTO-based 2DEGs. The second-harmonic resistance ($R_{xx}^{2\omega}$) as a function of in-plane magnetic field under 330-nm illumination and in the dark at 5 K. The nonreciprocal transport coefficient under 330-nm illumination also has the giant enhancement of three orders of magnitude, which further confirms the light-induced giant enhancement of nonreciprocal transport at the CZO/KTO interface.

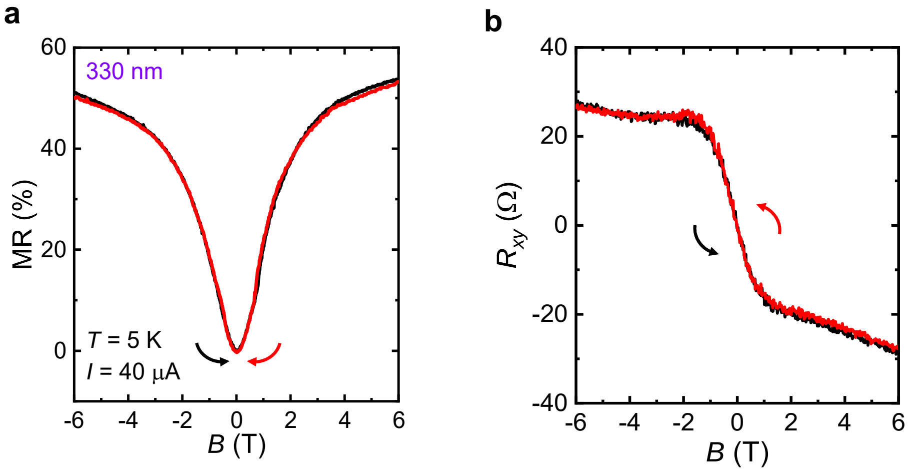

Supplementary Fig. 9. Hysteretic MR and R_{xy} measurements. a,b, MR and Hall curves under 330-nm irradiation with the applied out-of-plane magnetic field swept back and forth. We cannot observe any discernible hysteresis in both MR and Hall curves, indicating that light irradiation in our experiments cannot induce any ferromagnetic/spin-polarized 2DEGs at CZO/KTO interfaces.

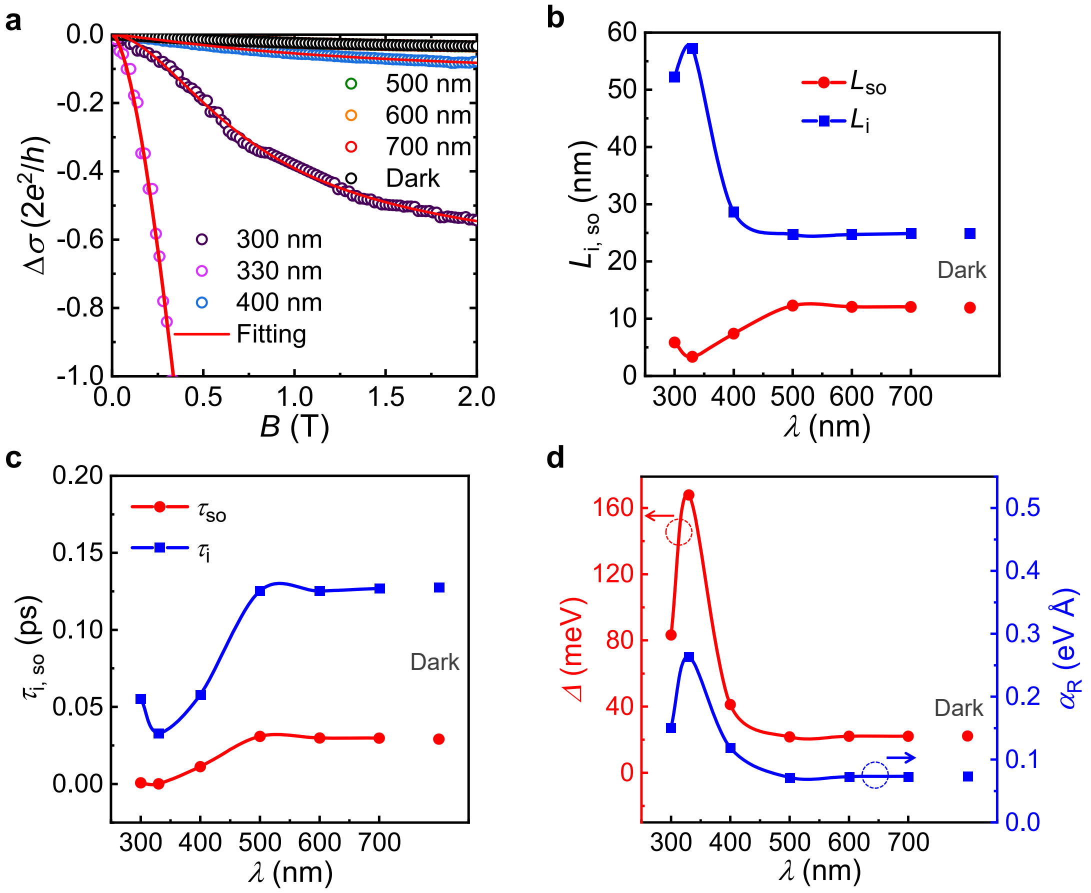

Supplementary Fig. 10. The magnetoconductance curves and the deduced other SOC-related parameters at various wavelengths at 5 K. a, The fitting curves of $\Delta\sigma$ according to the MF model at various wavelengths. This is the enlarged figure of **Fig. 3b**. **b-d**, The wavelength-dependent spin relaxation length (L_{so}) and dephasing length (L_i) in **b**, spin relaxation time (τ_{so}) and inelastic scattering time (τ_i) in **c**, and spin-splitting energy (Δ) and Rashba coefficient (α_R) in **d**, respectively. The corresponding parameters of the dark condition are also included in **b-d** for comparison.

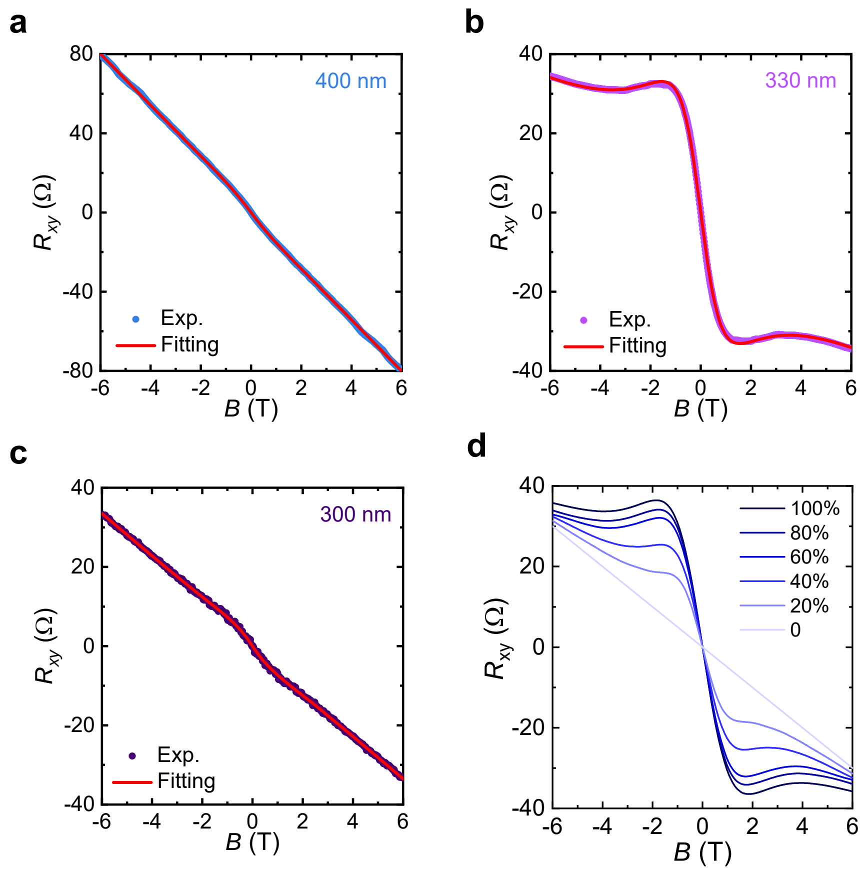

Supplementary Fig. 11. Hall curves perfectly fitted using the two-band model. **a-c**, The fitting curves of the Hall resistance at 400-nm, 330-nm and 300-nm illumination using the two-band model (equations (3) and (4) in the main text). Note that the kinks occurring at the Hall resistance at around ±1 T of 330-nm illumination in **b** are originated from the significant excitation of the second type of carriers with extremely high mobility at 330 nm. **d**, The Hall curves measured at various light powers normalized with that in **b**, indicating the clear excitation process from single type carrier (linear Hall resistance) to two types of carriers (nonlinear Hall resistance) with increasing light power.

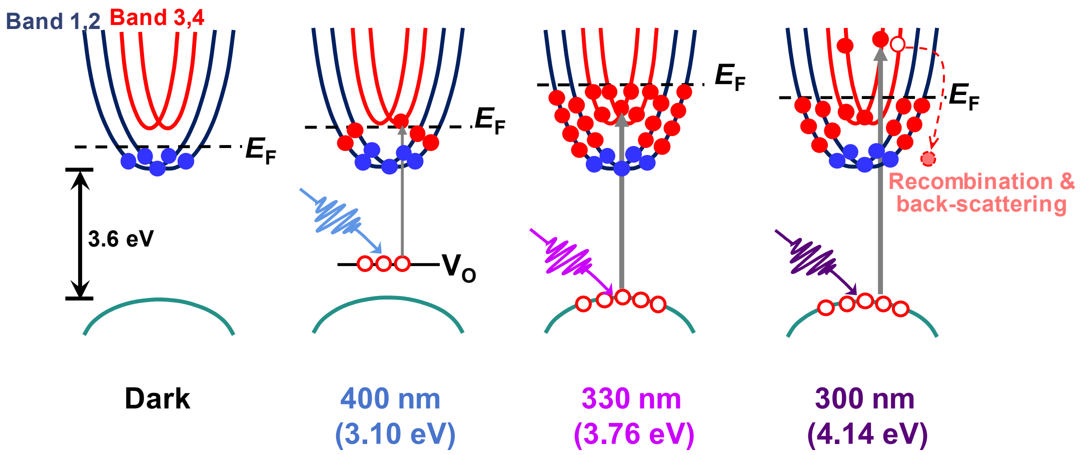

Supplementary Fig. 12. The physical mechanism of photocarrier excitation. In the dark, 2DEGs form at the CZO/KTO interface and there are abundant electrons in the potential well, as shown in Fig. 2c. Under the 400-nm illumination, electrons are excited from in-gap states of KTO to the CBM and transfer to the potential well. In this case, the intrinsic carriers increase a little and the second-type carriers emerge (grey arrows). Under the excitation of 330-nm light, abundant electrons are excited from the VBM to the CBM, inducing the considerable increase of both the intrinsic carriers and the second-type carriers as well as the E_F-level upward shift. However, under the excitation of 300-nm light, the total carrier density cannot be increased further. In this case, electrons can be excited to the higher subbands (above Band 3,4) of the t_{2g} conduction band. The higher-energy photoelectrons lead to increased interactions between electrons and the lattice (phonons), which results in a longer time for the electrons to return to a lower energy state. During this enhanced relaxation process, more photogenerated electrons would experience a notable competition between the weak localization and weak antilocalization in addition to the likely recombination of photogenerated electrons and holes. Hence, compared to the condition under 330-nm illumination, the final carrier density responsible for conductivity decreases and E_F level moves downward.

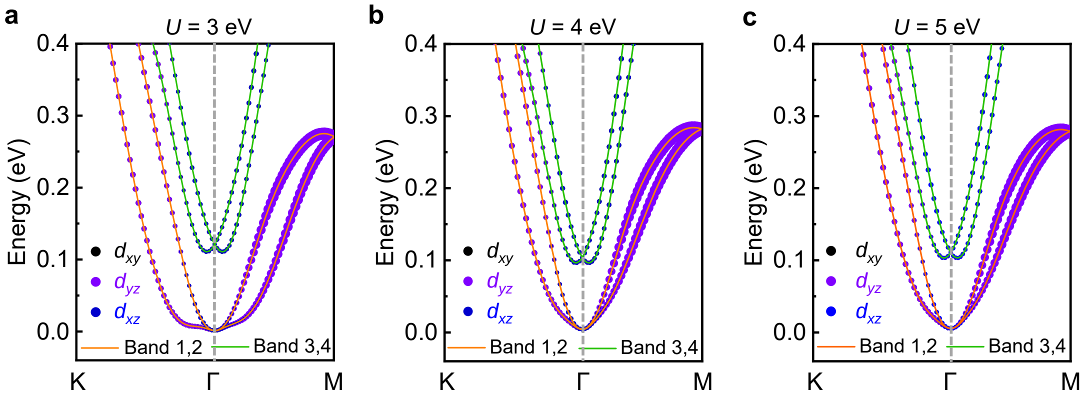

Supplementary Fig. 13. The band structures from first-principles calculations plus Hubbard *U*. a-c, The calculated electronic band structures (Band 1-4, orange and green lines) and the weight of d_{xy}, d_{yz} and d_{xz} (black, purple and blue circles) orbitals of 12-Ta layers of KTO (111) surface for $U = 3$, 4 and 5 eV, respectively. The weight of the orbitals is expressed by the size of the corresponding circles. There is no significant difference among calculated electronic band structures of KTO under different Hubbard U values, indicating that light only impacts on the location of E_F, thereby influencing the Rashba SOC strength as well as the nonreciprocal transport.
