# Supplementary material for: Light-induced giant enhancement of nonreciprocal transport at KTaO3-based interfaces
Source: Nat Commun. 2024 Apr 6;15:2992. doi: 10.1038/s41467-024-47231-6 (PMC10998845; doi:10.1038/s41467-024-47231-6)
Supplement: Supplementary file 3 — Description of Additional Supplementary Files [file 41467_2024_47231_MOESM3_ESM.pdf]

Description of Additional Supplementary File

File Name: Supplementary Data 1

Description: The atomic coordinates of the optimized computational models for $U = 3$, 4 and 5 eV.
